# Identifying factors contributing to social vulnerability through a deliberative Q-Sort process: an application to heat vulnerability in Taiwan

**DOI:** 10.1007/s11069-022-05280-4

**Published:** 2022-03-01

**Authors:** Leslie Mabon, Wan-Yu Shih

**Affiliations:** 1 School of Engineering and Innovation, The Open University, Milton Keynes, UK; 2 Department of Urban Planning and Disaster Management, Ming-Chuan University, Taipei, Taiwan

**Keywords:** Expert elicitation, Heat hazard, Q-Methodology, Taiwan, Vulnerability

## Abstract

Extreme heat events are gaining ever more policy and societal attention under a warming climate. Although a breadth of expertises are required to understand drivers of vulnerability to hazards such as extreme heat, it is also acknowledged that expert assessments in group settings may be subject to biases and uneven power relations. In this Technical Note, we outline a structured deliberative process for supporting experts to work collaboratively to assess social vulnerability to a climate-related hazard, in this case extreme heat in Taiwanese cities. We argue that adapting elicitation approaches such as Q-Methodology for use in collaborative settings can help to organise expert discussion and enable dialogue and mutual learning, in a way that supports consensus-building on vulnerability assessment. Outcomes from our collaborative assessments suggest elderly people living alone, elderly people over 75, pre-existing circulatory diseases and level of participation in community decision-making may all be notable drivers of heat vulnerability in the Taiwanese context. Methodologically, we argue that collaborative sorting exercises offer a way to embed local and experiential knowledges into assessments of available evidence, but that strong facilitation and additional checks are necessary to ensure an inclusive process that reflects the diversity of perspectives involved.

**Supplementary Information:**

The online version contains supplementary material available at 10.1007/s11069-022-05280-4.

## Introduction and rationale

Policy and societal awareness of the risks posed by extreme heat under a changing climate are growing. The increased frequency of high-profile heat incidences such as the ‘heatdome’ events in north-west USA and Canada in summer 2021 led *Nature* to state that urban governors and planners need to do more to address extreme heat within climate adaptation strategies (Nature 2021). The dangers from heat arise not only from discrete extreme heat events (i.e. acute heat lasting several days); but also from gradual rises in average temperature that lead to an overall increase in exposure to heat outside of recognised heat events (i.e. chronic heat that constitutes a slower-onset hazard) (Oppermann et al. 2021). Hazard from both extreme and chronic heat events is particularly serious in cities in the Tropics, which face a disproportionate warming trend as well as enhanced heat island effects with dense and rapid development (Ramsay et al. 2021).

As with other climate change risks, some sections of society may be at greater risk from extreme heat than others (Shih et al. 2022). Social vulnerability and responses to heat requires knowledges from a range of academic disciplines to understand comprehensively. Medical and public health expertise can give insight into physiological and demographic factors that may increase individuals’ risk from heat (e.g. Watts et al. 2019). Yet knowledge from areas such as sociology, urban planning, and social policy can build a richer understanding of factors that inform people’s ability to reduce their risk from heat, such as community connectedness during extreme heat events (Klinenberg 2002), priority for cooling measures in urban planning processes (Byrne et al. 2016), capacity to access risk communication information (Sampson et al. 2013) and effectiveness of public policies to reduce heat risk (Boeckmann 2016).

The purpose of this technical note is to demonstrate an approach through which different expertises can be brought together to deliberatively assess and localise a body of underlying peer-reviewed evidence, in order to develop an index of heat vulnerability factors appropriate to a locality. In doing so, we build on existing scholarly interest in systematic and evidence-driven participatory processes to synthesise knowledge from experts in situations of high uncertainty (e.g. Raikes and McBean 2017; Räsänen et al 2019). We explore our technique through the issue of heat vulnerability for cities in Taiwan, a country predicted to face notable heat risk but one where empirical evidence on societal drivers of heat vulnerability is limited (Shih and Mabon 2021).

## Structured expert assessments for climate risk

Comprehensive understanding of risk from heat—and indeed climate risks more broadly—requires expertise on the influence of a breadth of factors to be included and evaluated within vulnerability assessment processes (Cutter et al 2014). Moreover, different factors will influence vulnerability differently across country contexts (Turek-Hankins et al. 2021), meaning a pool of local interdisciplinary expertise is necessary to inform locally-appropriate strategies. Local governments themselves are looking to expert panels and committees to support their climate responses, as illustrated in the Edmonton Declaration which calls on cities to adopt science-based decision-making or even appoint chief scientists (City of Edmonton 2018).

However, within expert-driven decision-making in an environmental policy context, certain disciplinary groups of experts can dominate and steer policy recommendations towards their preferred issues and strategies (Finewood 2016; Lo and Chen 2019). Morgan (2014) argues that when expert assessments for issues including climate change risk are carried out in group settings, discussions often proceed informally with little acknowledgement of the potential influence of biases within groups. The danger is therefore that multi-disciplinary processes for collaboratively assessing climate risks may be open to power imbalances and influence, which may lead to assessments and recommendations which are not as comprehensive or balanced as one may anticipate.

Partly in response, a breadth of techniques have been developed with the aim of providing a systematic and structured process to capture and synthesise expert assessments. In this Technical Note, we explore and develop one such approach which can be applied in an expert context—Q-Methodology. In a Q Study, participants sort a series of statements into a grid of numbered columns running from positive through to negative integers, sorting the statements in order from those they most agree with to those they least agree with (Eden et al. 2005). Q-Methodology has been used in an environmental context to identify issues of priority and concern in, for example, non-governmental organisation (NGO) attitudes to environmental science in the UK (Eden et al. 2005); stakeholder responses for flood management on the Scotland-England border (Forrester et al. 2015); and municipal managers’ responses to disaster risk in British Columbia, Canada (Raikes and McBean 2017). In these contexts, ‘expertise’ can be seen to encompass not only techno-scientific expertise, but also expertise gained through practice and experience.

Examples of the application of Q-Methodology to heat vulnerability in the literature are more limited. An analogue may, however, be found in Räsänen et al. (2019) who use a similar structured technique—Delphi Method—with expert respondents to assign relative weightings and identify uncertainties and areas of contention among socio-economic drivers of heat vulnerability in Helsinki, Finland. Räsänen et al. use these weightings to develop maps showing areas of high vulnerability within the city, and also locations of high uncertainty where there is divergence between expert assessments. Räsänen et al. explain that understanding expert consensus and divergence in this way is valuable in helping policy-makers to understand the relative drivers of vulnerability, and also the probability of vulnerability, in a locality. Schlosberg et al. (2017), looking at heat in combination with other climate-related hazards, combine Q-Methodology with a deliberative citizens’ panel to create citizen-led recommendations and priorities for climate adaptation policy in Sydney, Australia. What is notable about the work of Schlosberg et al. is that it combines Q-Methodology with deliberative processes—participants completing individual Q-Sorts and then engaging in dialogue and deliberation—under the aim of building consensus on areas of priority for a complex and challenging area such as climate risk.

## Context: Taiwan and urban heat

Taiwan has experienced a significant warming trend over the last hundred years. In the past century, the average annual temperature in the lowland areas has risen by 1.6 degrees Celsius. Months considered ‘summer’ have increased to 120–150 days; whereas months considered ‘winter’ have reduced to 20–40 days (TCCIP 2021). In the warm season between 2000 and 2014, daily maxima for heat-related hospital admissions and emergency visits have reached 1533 and 73 people, respectively (Cheng et al. 2019). Chung et al. (2009) indicate that every 1 °C increment above 36 °C in air temperature will lead to an increase of heat health risk by 5% in Taiwan. According to the Taiwan Climate Change Projection Information and Adaptation Knowledge Platform (TCCIP), in the worst-case global warming scenario (SSP5-8.5), extreme high temperatures (above 36 °C) will be reached 48.1 days per year by the end of the century, and cities will suffer the most (TCCIP 2021).

Taiwanese cities have a dense development pattern and a humid subtropical climate, where extreme heat in summer is becoming a serious concern (Cheng et al. 2019; Shih and Mabon 2021). Taiwanese cities are hence indicative of the development and climatic conditions in which many people globally will experience extreme heat. Socially too, Taiwan may offer valuable insights for other nations globally (Shih et al. 2022). It is a comparatively new democracy, and one which has also embraced participatory decision-making in recent years in response to a breadth of social and environmental challenges. Taiwan has also won praise globally for its evidence-driven and rapid response to COVID-19. Yet Taiwan also has an ageing population (15.9% of the population are defined as elderly as of 2021), with a growing migrant workforce that has increased by 63.8% since 2010 (DGBAS 2021) and is arguably not well-recognised within the country’s social policies. The social pressures and political landscape faced by Taiwan, of democratisation and digitalisation at the same time as socio-demographic change (see e.g. Dunch and Esarey 2020), hence yield insight for other locales globally for making sense of heat vulnerability at times of socio-political change.

## Method

Our aim is to develop and trial a method that combines the value of deliberative approaches in reconciling a breadth of knowledges and expertises when approaching a complex topic (Forrester et al. 2015; Räsänen et al. 2019) with the acknowledgement that biases and power relations may still influence the outcomes of evenly seemingly ‘science-led’ deliberative processes if left unchecked (Morgan 2014). In particular, we sought to evaluate the assertion of Morgan (2014: p. 7183) that “the performance of consensus expert panels might be improved if panel members first performed individual elicitations before they begin their group deliberations”; and also the view of Räsänen et al. (2019) that the learning process that happens during vulnerability assessment may be as valuable as the final outcome.

To guide the discussion and ranking exercises, factors driving social vulnerability to heat were identified through review of existing peer-reviewed literature, conducted by searching Web of Science for the terms ‘heat vulnerability’ AND ‘social’ OR ‘society’. The aim of the review was to identify factors which had been found to influence heat vulnerability in previous studies, particularly those undertaken in Asian cities. The findings of the review were then condensed into 36 statements relating to heat vulnerability, which formed the basis of a Q-Sorting exercise (statements are listed in the Supplementary Data, and summarised in Figs. 5, 6, 7, 8). Where our approach differs to more conventional applications of Q-Methodology is that, rather than having participants conduct individual Q-Sorts which would then be analysed to identify ‘factors’ and shared views about a topic (e.g. Eden et al. 2005; Raikes and McBean 2017), we instead asked an expert panel to work together to collaboratively undertake a single Q-Sort. In doing so, we sought to achieve a final heat vulnerability ranking that was representative of the breadth of knowledges and expertises in the room. The intention was to draw on the deliberative and discursive qualities of the Q-Sorting process (Eden et al. 2005; Forrester et al. 2015) to act as a structured learning exercise where participants were able to debate and discuss their assessments of the relative priority of different factors and come to a consensus.

Expert participants were invited to attend a half-day in-person workshop on heat vulnerability in the Taiwanese context, held in Taipei. Experts were selected on the basis of their familiarity with the topic, as assessed by having peer-reviewed publications on heat, a strong media presence and/or government expert committee membership, and were recruited to cover a breadth of academic disciplines and policy/practice sectors. To broaden the pool of experiences and include local and experiential knowledges with which the researchers may not have been familiar, an open call for application to participate was also held. In total, this process yielded eighteen participants (see Supplementary Data)—eight academics (public health, urban planning, geography and architecture), six consultants (built environment, landscape, and urban planning), one architect, and three people from civil society organisations.

**Fig. 1:**
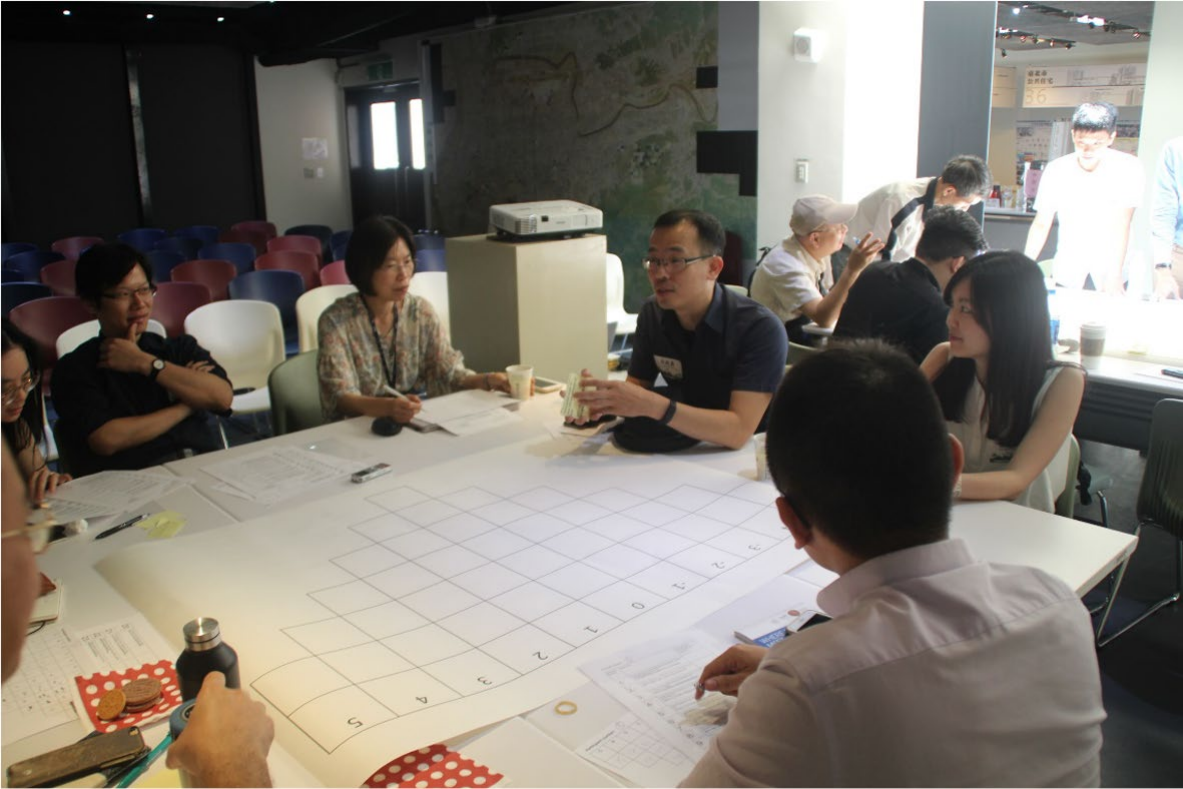
Participants discussing their relative agreement with different statements in small groups after completing the individual Q-Sorts

**Fig. 2:**
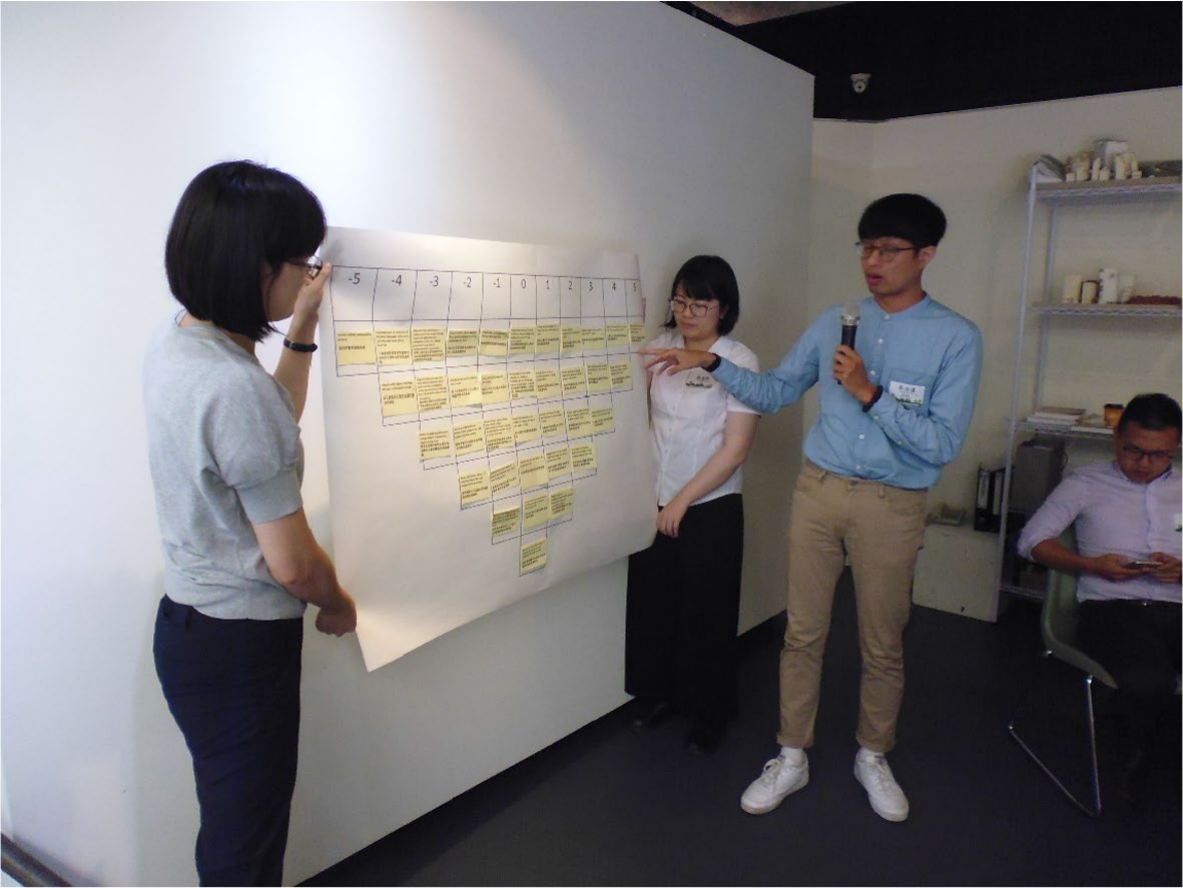
Small groups presenting their first collaborative sort to the wider group, as a stimulus for whole group discussion and the final Q-Sort

**Fig. 3:**
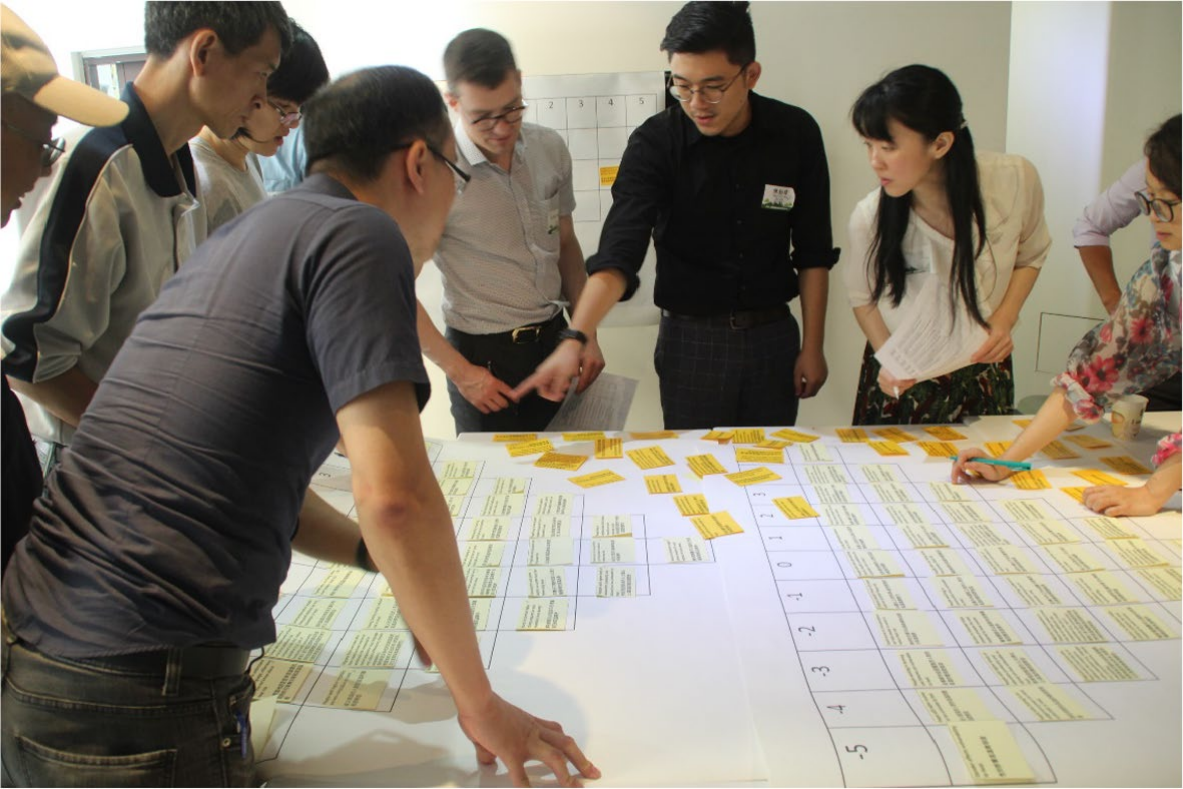
The entire expert group working together, with facilitation from the research team, to come to consensus on the final Q-sort

Following a short introductory presentation from the research team and a talk from an invited expert on the experience of developing vulnerability indicators in an international setting, participants were given individual Q-Sorting grids and a list of statements, and asked to work individually to sort the statements according to the agreement or importance they wished to assign a given statement. The grids were arranged in shape of an inverse pyramid, where the middle column was assigned the value of 0 to allow the position of neutral statement. Statements were then allocated to columns to the right with a value of between 1 and 5 to show increasing agreement/importance; or to the left with a value of between − 1 and − 5 to show increasing disagreement or perceived unimportance (see Fig. 1). Asking participants to complete individual Q-Sorts at the start of the workshop served the purpose of (a) giving participants time to consider the statements and to develop their argumentation before group discussion; and (b) enabling the researchers to assess the extent to which individual Q-Sorts match up to the subsequent collaborative sorts (reflecting the suggestion of Morgan (2014)). After filling in the individual sorts, participants were divided into two sub-groups, where they worked collaboratively to discuss and produce a small group Q-Sort (using the individual Q-Sorts as a stimulus for discussion). The two sub-groups then presented their Q-Sort and associated rationale to the whole group. With the lead researchers facilitating the process, the whole expert group of 18 people then worked to come to consensus on the final sorting and ranking of the 36 statements (see Figs. 1, 2, 3). Participants were asked to sort the statements according to the extent to which that they agreed the statement represented a driver of heat vulnerability.

**Fig. 4:**
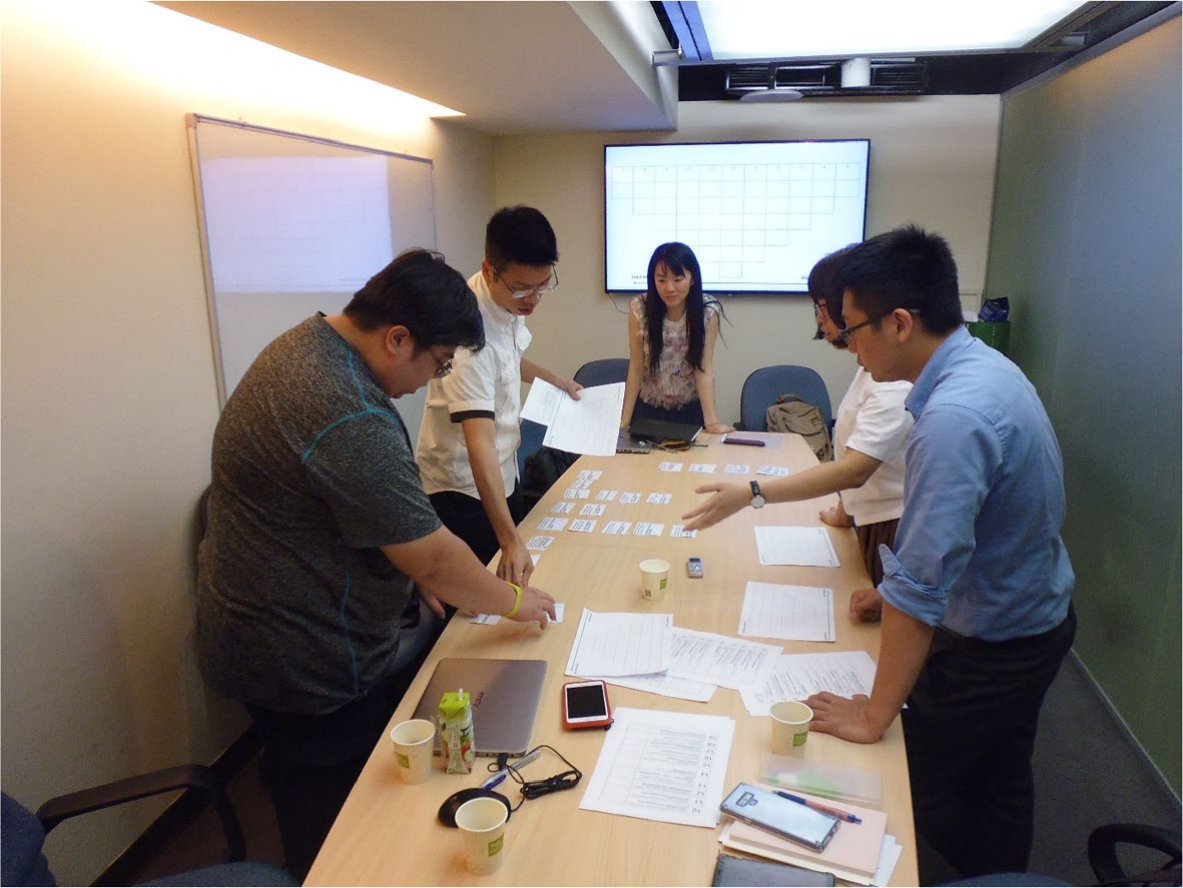
Smaller group of professional students working together under facilitation to collaboratively sort the statements according to the Q-Sort grid pattern

To further evaluate the method and consider its suitability for integrating local and experiential knowledges as well as technical expertise, the process was later repeated with a smaller group of professional students with different professional backgrounds (see Fig. 4) who were studying for a postgraduate degree in the Urban Planning and Disaster Management department of Ming Chuan University in Taiwan (see Supplementary Data).

## Results

Whilst our main focus is evaluating deliberative Q-Sorting as a method, it is also worth sharing our key findings. We report the results from the Q-Sorts with descriptive statistics of mean value and standard deviation (SD) for the values assigned to each of the 36 statements by the entire group; and compare these statistics between sub-groups.

### Expert group

**Fig. 5:**
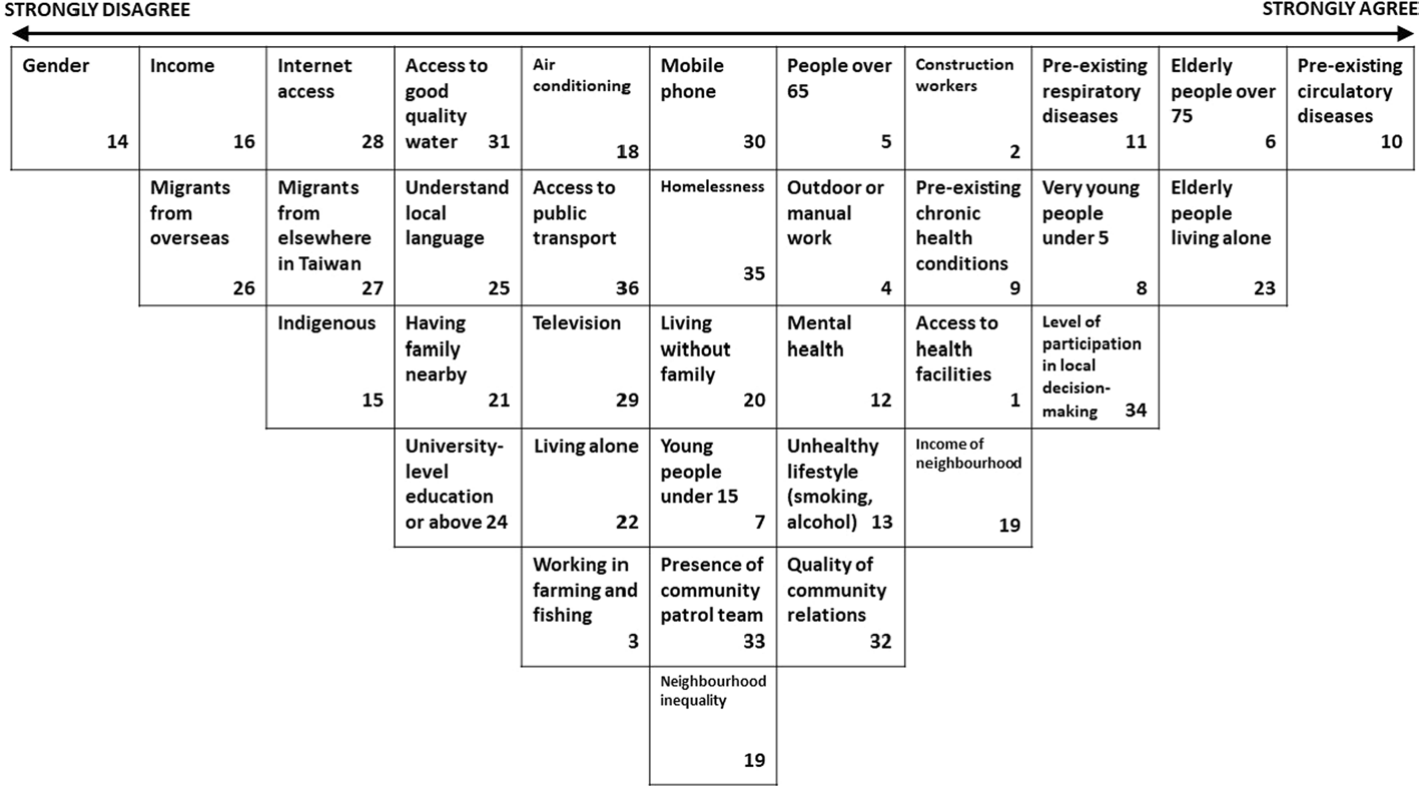
Whole group sort by Taipei experts (Note: the number in each box refers to the number assigned to each statement for ease of sorting, and are included here to enable cross-comparison.)

In the final sort conducted by the whole group (Fig. 5), the statement *People with pre-existing circulatory diseases are at greater risk in heat* was sorted as having the highest agreement (+ 5), followed by *Elderly people living alone are at risk in heat* (+ 4) and *Elderly people (over 75 years) are at great heat risk* (+ 4). Conversely, the statement *Gender affects vulnerability to heat* (− 5) was sorted as being least agreed on by the experts, followed by *People with higher incomes are less vulnerable to heat* (− 4) and *Migrants from overseas who have newly arrived may be at additional risk compared to the native population* (− 4).

Across the individual Q-Sorts (Table 3), the statements sorted highest on average were *Very young people (under 5 years old) are vulnerable to heat* (+ 1.89); *Elderly people (over 75 years) are at great heat risk* (+ 1.67); and *Communities with patrol teams can counter heat risks from living alone*; *People with pre-existing circulatory diseases are at greater risk in heat*; and *Elderly people living alone are at risk in heat* (all + 1.56). The statements ranked lowest on average were *People who belong to an indigenous group are more vulnerable to heat* (− 2.67); *Gender affects vulnerability to heat* (− 2.00); and *Access to television helps citizens prepare for and respond to heat events* (− 1.67).

The statements with the largest difference in assessment between participants (i.e. those with the largest standard deviation from the mean) (see Supplementary Data) were *People living in communities where there is more participation in decision-making may be better connected and so at less risk from heat* (SD 3.24); *People working outdoors or in manual work are at particular risk* (SD 3.18); and *Access to mobile phone helps citizens prepare for and respond to heat events* (SD 2.88).

Key points to note here are that statements which recorded high levels of agreement in the collaborative group sort were generally also ranked highly in the individual sorts. Similarly, statements which the group gave low agreement and hence low priority to in the collaborative sort also tended to be given low priority in the individual sorts. Although the statement with the highest disagreement between participants (i.e. the biggest difference in opinion) *People living in communities where there is more participation in decision-making may be better connected and so at less risk from heat* was sorted comparatively highly (+ 3), other statements with high standard deviation tended to be sorted by the whole group to near the middle positions (i.e. neither strongly agree nor disagree) on the grid.

#### Expert sub-groups

**Fig. 6:**
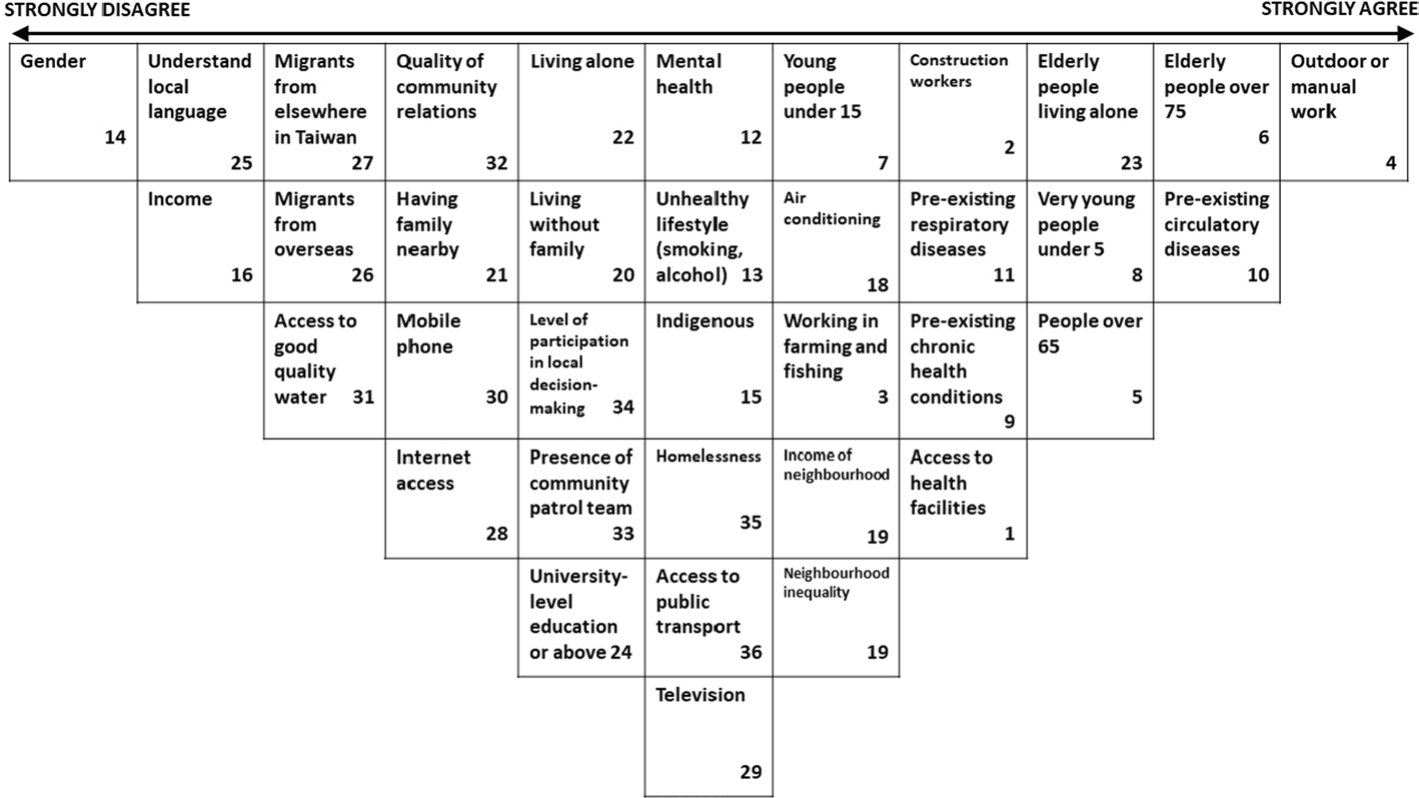
Group sort by Taipei expert sub-group 1

It is also worth briefly exploring the outcomes from the sub-groups. *Sub-Group 1* (Fig. 6) in their small group sort agreed most with the statements *Elderly people living alone are at risk in heat* (+ 5); *People living in communities where there is more participation in decision-making may be better connected and so at less risk from heat* (+ 4); and *People with pre-existing circulatory diseases are at greater risk in heat* (+ 4). Sub-group 1 disagreed most with the statements *Gender affects vulnerability to heat* (− 5); *People with higher incomes are less vulnerable to heat* (− 4); and *People who belong to an indigenous group are more vulnerable to heat* (− 4).

Among the individual Q-Sorts of those participating in Sub-Group 1, the statements gathering strongest agreement were *Communities with patrol teams can counter heat risks from living alone* (+ 1.8); *Access to television helps citizens prepare for and respond to heat events* (+ 1.6); and *People who are homeless are at greater risk*; *Living with family reduces risk in heat*; and *Good access to medical facilities (hospitals, clinics) mitigates against heat risk* (all + 1.4). Those with strongest disagreement on average were *People who belong to an indigenous group are more vulnerable to heat* (− 3.8); *Gender affects vulnerability to heat* (− 1.8); and *People with mental health problems may be subject to additional stress during extreme heat* (− 1.6). The statements with the biggest difference in opinion between experts for Sub-Group 1 were *Having access to an air conditioner reduces heat vulnerability* (SD 3.7); *People living in communities where there is more participation in decision-making may be better connected and so at less risk from heat* (SD 3.56); and *Elderly people living alone are at risk in heat* (SD 3.27)*.*

**Fig. 7:**
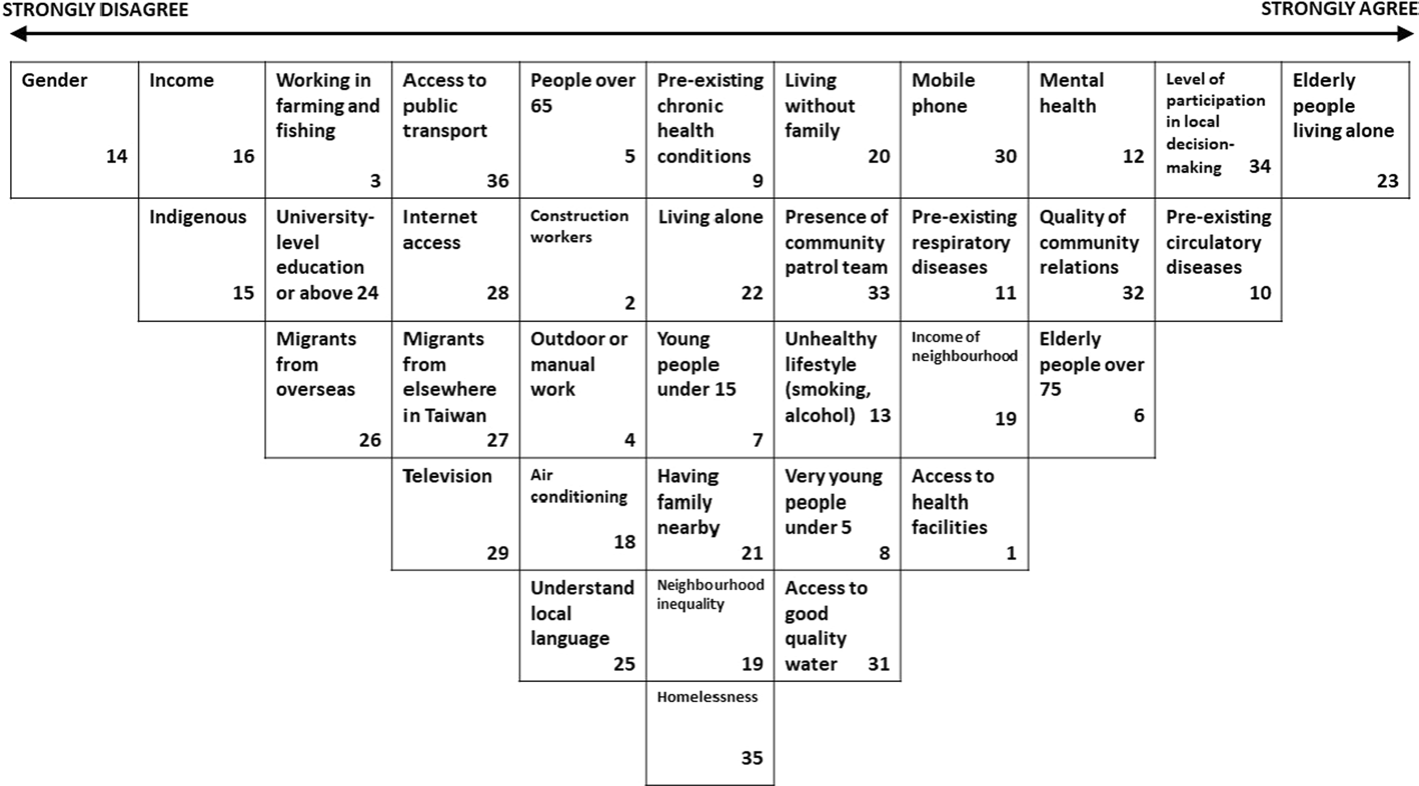
Group sort by Taipei expert sub-group 2

*Sub-Group 2* (Fig. 7) gave the most positive scores to *People working outdoors or in manual work are at particular risk* (+ 5); *Elderly people (over 75 years) are at great heat risk* (+ 4); and *People with pre-existing circulatory diseases are at greater risk in heat* (+ 4). This group collaboratively gave the most negative scores to the statements *Gender affects vulnerability to heat* (-5); *It is important to understand the local language, otherwise you may not know about heat risk* (-4); and *People with higher incomes are less vulnerable to heat* (− 4).

In the individual Q-Sorts of experts assigned to Sub-Group 2, the highest mean level of agreement was with the statements *People working outdoors or in manual work are at particular risk* (+ 3.18); *Elderly people living alone are at risk in heat* (+ 2.83); and *Elderly people (over 75 years) are at great heat risk* (+ 2.69).The lowest mean level of agreement was with the statements *Access to internet helps citizens prepare for and respond to heat events* (− 2.5); *Gender affects vulnerability to heat* (− 2.25); and *People with higher incomes are less vulnerable to heat* (− 1.75). The statements with biggest difference in opinion for this group were *Access to mobile phone helps citizens prepare for and respond to heat events* (SD3.4); *Access to internet helps citizens prepare for and respond to heat events* (SD3.3); and *Gender affects vulnerability to heat* (SD3.2)*.*

### Disaster management professionals

**Fig. 8:**
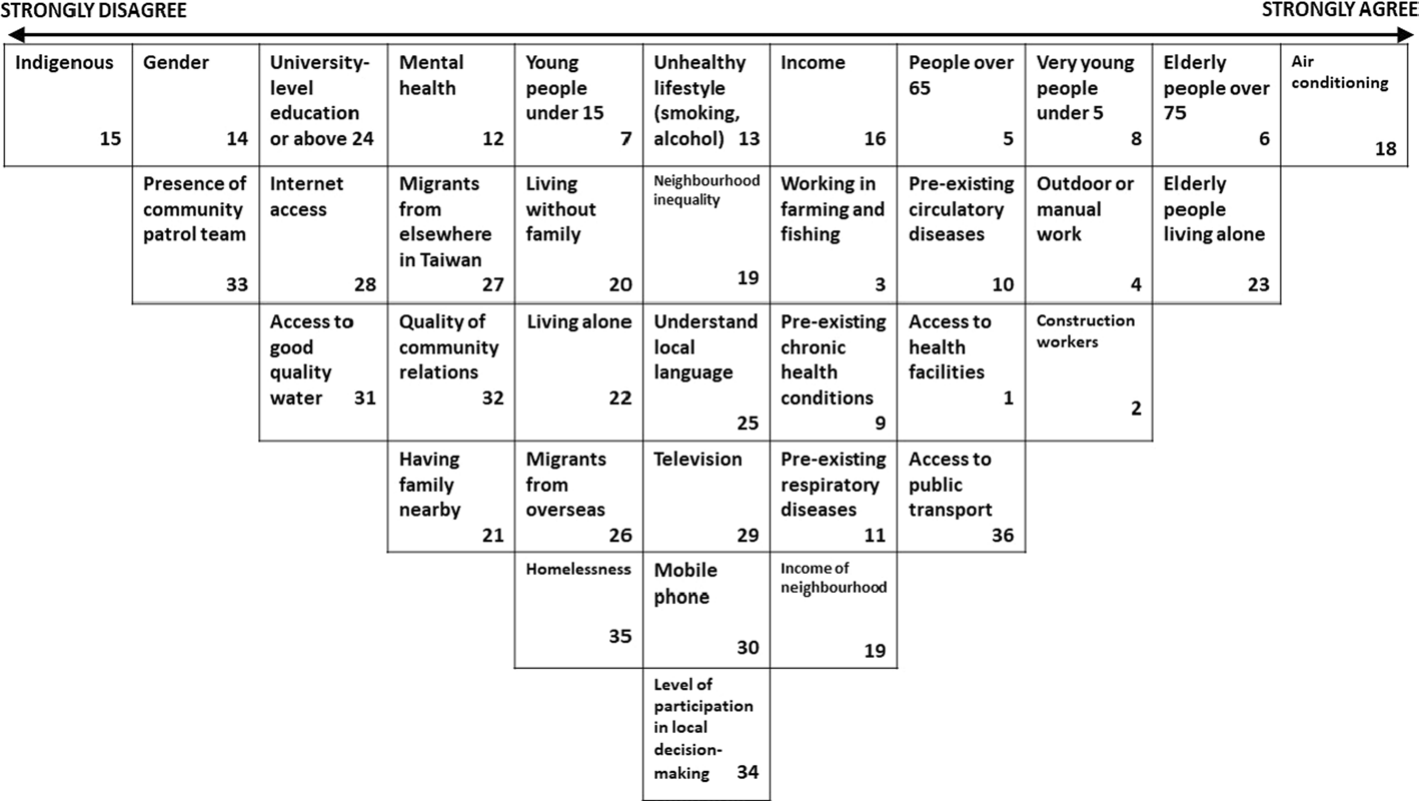
Group sort by Taipei disaster management professional students

As noted in the Method section, the exercise was also repeated with a small group of professional students from an urban planning and disaster management department (Fig. 8). This group agreed collaboratively most with the statement that *having access to an air conditioner reduces heat vulnerability* (+ 5); *Elderly people living alone are at risk in heat* (+ 4); and *Elderly people (over 75 years) are at great heat risk* (+ 4). The group collaboratively disagreed most with the statements *People who belong to an indigenous group are more vulnerable to heat* (− 5); *Gender affects vulnerability to heat* (− 4); and *Communities with patrol teams can counter heat risks from living alone* (− 4).

This disaster management group reported highest agreement in their individual Q-Sorts with the statements *Having access to an air conditioner reduces heat vulnerability* (+ 4.25); *People working outdoors or in manual work are at particular risk* (+ 2.75); and *Elderly people (over 75 years) are at great heat risk* (+ 2.75). Strongest disagreement was reported with the statements *Gender affects vulnerability to heat* (− 3.5); *People who belong to an indigenous group are more vulnerable to heat* (− 3); and *Communities with patrol teams can counter heat risks from living alone* (− 2.5). The biggest differences in opinion came with the statements *Communities where people have good relations are less vulnerable to heat* (SD3.32); *People living in communities where there is more participation in decision-making may be better connected and so at less risk from heat* (SD3.16); and *People with higher incomes are less vulnerable to heat* (SD2.94).

## Discussion

The main aim of this technical note is to evaluate collaborative Q-Sort as a structured deliberative method to guide collaborative expert assessment of social vulnerability to heat and wider environmental hazards. However, it is also worth briefly reflecting on the results themselves and their methodological implications.

### Key findings and relation to existing literature

Across all groups, *elderly people living alone, elderly people over 75,* and *outdoor workers* were assessed as important drivers. This is consistent with existing literature in Taiwan (see e.g. Lin and Chan 2009; Cheng et al. 2019; Shih and Mabon 2021). Among the heat vulnerability expert groups, *pre-existing circulatory diseases* and *level of participation in community decision-making* were also assessed as important drivers of heat vulnerability. Conversely, across all groups, *gender*, *personal income*, *indigenous status, and migrant worker status* were assessed as having less effect on heat vulnerability in Taiwan. This differs from what has been seen in empirical peer-reviewed literature, which has argued for the importance of such factors (e.g. Wu et al. 2011; Wang and Lin 2015; Shih et al. 2022). Across the individual assessments, the influence of *level of participation in community decision-making*, whilst ranked highly in the final collaborative sorts, was a particular source of differing opinion.

The relatively high priority of age and health status in our Q-Sorts might reflect the number of public health experts involved in the process. Socio-economic factors, such as gender, race, education, and income may have been assigned higher priority if experts from a wider range of disciplines, income diversity, associated research experiences or indigenous groups had participated in the workshops. This is notable as, in an expert elicitation process for heat vulnerability in Taiwan based on survey-based methods, Shih and Mabon (2021) similarly found experts giving gender and indigenous status low priority, despite peer-reviewed evidence from Taiwan and internationally suggesting these factors can be important drivers of vulnerability to natural hazards (e.g. Huang 2018). We now turn to the methodological implications of these findings.

### Structured deliberative vulnerability assessment: reflections

Across all groups, there was generally good consistency between the mean rankings participants made in their own Q-Sorts carried out independently at the start of the workshop, and the final rankings reached deliberatively by the whole group working together. This indicates that a collaborative and deliberative ranking exercise may produce a final vulnerability assessment that broadly reflects and balances the full range of assessments and expertises in the room. Structured approaches to expert deliberation such as the collaborative Q-Sort we have outlined here may hence go some way to addressing the concern of Morgan (2014) about biases going unchecked in informal expert assessment discussions, and the wider points in the literature about specific individuals or communities exerting disproportionate influence over environmental policy fora (Lo and Chen 2019). Our approach may also demonstrate in practice the value of panel members first performing individual elicitations before discussions begin in the way Morgan suggests. Indeed, in our deliberative exercises, having experts perform their own individual elicitations beforehand meant they had time prior to the commencement of discussion to consider their own assessment, and were then able to refer to their own assessments and judgements to guide the discussion.

Nevertheless, whilst the sub-group discussions also returned collaborative sorts that were broadly consistent with the individual assessments by their participants, it is notable in each case that statements recording high levels of disagreement within the sub-group (levels of participation and elderly people living alone for Group 1; Gender for Group 2) obtained either very high or very low scores in the collaborative sort. This may reflect our observation that in the sub-groups, which were self-organising and not strongly facilitated, one or two dominant individuals were able to dominate the discussion and could simply ‘take charge’ and decide the priority for statements where there was significant disagreement. Reflecting the view of Räsänen et al. (2019) that the dialogue and learning process between experts may be as valuable an outcome of a collaborative vulnerability mapping activity as the final assessment itself, our observations thus show the importance of strong facilitation to ensure that one or two experts do not dominate a discussion on priority factors, and that all participants are able to contribute and learn from each other. Competent facilitation may be especially important in smaller groups, such as our sub-groups, where one or two voices may feel more comfortable to dominate.

At the same time, however, even in a deliberative situation an expert assessment can only be as effective as the breadth of expertises in the room. As above, the high prioritisation of demographic, socio-economic and health factors in our Q-Sorts may reflect the number of public health and urban planning experts involved in the process, and the issues that are usually emphasised in research into heat hazard in their own disciplines. Accordingly, just as the importance of diversity within citizen-focused deliberation is widely recognised, our insights build on the assertion of Roberts et al (2020) that attention ought to be paid to diversity in disciplinary background, socio-economic status and life experience of experts in deliberative processes.

Our final comment relates to the value of a structured deliberative vulnerability assessment process in integrating local and experiential knowledges of vulnerability alongside technical and scientific judgements. Jonsson and Lundgren (2015) have argued that municipal planners, health care workers and citizens themselves may have extensive knowledge of drivers of heat vulnerability in a locality, but that such knowledge rarely has a significant influence on policy. Similarly, Turek-Hankins et al. (2021) hold that knowledge of locally-appropriate heat adaptation strategies and vulnerability drivers may be held in traditional knowledge or in ‘grey literature,’ outside the body of peer-reviewed scientific evidence on heat. This was apparent in our research too. In the professional student group, for example, respondents brought in professional experiences from their roles in areas such as the postal service to argue for the importance of considering outdoor workers in addition to those in primary industries (Lin and Chan 2009). What we have tried to demonstrate in this report is an approach that allows different *kinds* of expert knowledge—encompassing different academic disciplines, practice-based knowledge, policy expertise, and local and experiential knowledge—to be used to evaluate the relative significance of different vulnerability drivers to local contexts, such as Taiwan, where there may be limited locally-specific empirical research to date.

As well as being a research exercise, structured deliberative processes for assessing heat vulnerability may therefore be useful for local decision-makers to run as a means of developing evidence-informed policy and plans for heat hazard. However it is important that such exercises (a) contain a good diversity of expertise, including local knowledge and embodied/practice-based knowledge; (b) are well facilitated to ensure the final outcomes reflect the range of assessments in the room; and (c) pay attention to the areas of greatest disagreement between experts as well as the areas of most agreement on vulnerability drivers.

## Conclusion

In this technical note, we have explored the potential for the adaptation of structured elicitation tools such as Q-Methodology to be used in a deliberative setting, through the case of social heat vulnerability assessment by experts in Taiwan. Whilst approaches such as Q-Methodology are normally undertaken by individuals and then analysed by researchers to identify common themes and factors, we have argued that adopting the approach taken by elicitation methods in this way can help to structure collaborative expert assessments. In turn, the dialogic nature of collaborative and deliberative sorting can enable mutual learning and help to reach consensus among experts from different disciplinary bases. Involving experts from outside of academia in a deliberative process of this nature also enables the group to weigh up existing available evidence in light of local and experiential knowledge as well as scientific knowledge. Nonetheless, the case of heat vulnerability assessment in Taiwan also shows there is a need for capable facilitation to ensure that all participants are able to engage within the consensus-building process, and that supplementing structured deliberative processes with individual elicitations may be important in helping to identify biases or disagreements within the final consensus-based assessment.

## Supplementary Information

Below is the link to the electronic supplementary material.

Supplementary file1 (PDF 24 KB)

## Data Availability

For reasons of data protection and privacy, the empirical data on which this technical note is based is not publicly available. However, reasonable requests will be considered by the authors.
