# Supplementary material for: Identifying factors contributing to social vulnerability through a deliberative Q-Sort process: an application to heat vulnerability in Taiwan
Source: Nat Hazards (Dordr). 2022 Mar 1;112(3):2609–23. doi: 10.1007/s11069-022-05280-4 (PMC8885317; doi:10.1007/s11069-022-05280-4)
Supplement: Supplementary file 1 — Supplementary file1 (PDF 24 KB) [file 11069_2022_5280_MOESM1_ESM.pdf]

Technical Note: Identifying factors contributing to social vulnerability through a deliberative Q-Sort process: an application to heat vulnerability in Taiwan (DOI: 10.1007/s11069-022-05280-4)

Supplementary Data

Table 1: expert workshop participants

**Group 1**	
Academic (public health)	Consultant (built environment)
Academic (architecture)	Architect
Academic (public health)	NGO (landscape planning)
Academic (architecture)	Civil society (culture park volunteer)
Academic (geography)	Consultant (urban planning)
**Group 2**	
Academic (urban planning)	Consultant (landscape planning)
Academic (public health)	Consultant (engineering)
Academic (geography)	Consultant (urban planning)
Consultant (built environment)	NGO (urban planning)

Table 2: disaster management professional workshop participants

**Disaster Management Professionals**	
Postal service management	IT
Real estate	Student dormitory manager

Table 3: individual Q-Sort rankings for whole expert group and small groups

Statement	Mean Rank (Whole Group)	Mean Rank (Small Groups) (Top = Grp 1; Bottom = Grp 2)	Standard Deviation (Whole Group)	Standard Deviation (Small Groups) (Top = Grp 1; Bottom = Grp 2)
1. Good access to medical facilities (hospitals, clinics) mitigates against heat risk	1.00	1.4	2.18	2.61
		0.5		1.73
2. People working in construction are vulnerable to heat	0.56	0	2.01	2.55
		1.25		0.96
3. People working in farming and fisheries are vulnerable to heat	0.22	-1	2.49	2.55
		1.75		1.5
4. People working outdoors or in manual work are at particular risk	1.11	-1	3.18	2.45
		3.75		1.5
5. Elderly people (over 65 years) are at great heat risk	0.78	0	2.54	2.74
		1.75		2.22
6. Elderly people (over 75 years) are at great heat risk	1.67	0.6	2.69	3.13
		3		1.41
7. Young people (under 15 years old) are vulnerable to heat	-0.11	-0.4	1.96	0.89
		0.25		2.99
8. Very young people (under 5 years old) are vulnerable to heat	1.89	1.2	2.20	1.79
		2.75		2.63
9. People who have pre-existing chronic health conditions are at greater risk in heat	0.33	-0.2	1.32	1.48
		1		0.82
	1.56	0.8	2.60	2.95

10. People with pre-existing circulatory diseases are at greater risk in heat		2.5		2.08
11. People with pre-existing respiratory diseases are at greater risk in heat	-0.56	-1.2	2.24	1.48
		0.25		2.99
12. People with mental health problems may be subject to additional stress during extreme heat	-0.78	-1.6	2.22	1.82
		0.25		2.5
13. People with an unhealthy lifestyle (e.g. tobacco smoking, alcohol drinking) are more vulnerable to heat	-1.44	-1.2	2.01	1.48
		-1.75		2.75
14. Gender affects vulnerability to heat	-2.00	-1.8	2.50	2.17
		-2.25		3.20
15. People who belong to an indigenous group are more vulnerable to heat	-2.67	-3.8	2.00	1.64
		-1.25		1.5
16. People with higher incomes are less vulnerable to heat	-0.78	0	2.49	3
		-1.75		1.5
17. People living in low-income neighbourhoods are more vulnerable to heat	0.33	0	1.41	1.73
		0.75		0.96
18. Having access to an air conditioner reduces heat vulnerability	0.89	0.8	2.85	3.70
		1		1.83
19. People living in neighbourhoods with greater inequality are more vulnerable to heat	0.56	0.8	2.13	2.56
		0.25		1.71
20. Living with family reduces risk in heat	0.11	1.4	2.57	2.30
		-1.5		2.08
21. Having family nearby reduces risk in heat	0.44	1.2	1.81	1.92
		-0.5		1.29
22. People who are living alone have greater heat risk	-0.22	-0.8	1.48	1.64
		0.5		1
23. Elderly people living alone are at risk in heat	1.56	0.2	2.83	3.27
		3.25		0.5
24. People with higher levels of education (university and above) are less vulnerable to heat	-0.56	0.2	1.42	1.10
		-1.5		1.29
25. It is important to understand the local language, otherwise you may not know about heat risk	-0.56	0	2.79	2.95
		-1.25		1.5
26. Migrants from overseas who have newly arrived may be at additional risk compared to the native population (e.g. language and cultural differences, climate adaptability)	-0.56	0	1.67	2
		-0.5		0.96
27. Migrants from elsewhere in Taiwan who have newly arrived may be at additional risk compared to the city's native population (e.g. lack of familiarity with community)	-0.22	0.6	1.72	2.35
		-1.25		0.58
	-0.22	-1	2.68	2.07

28. Access to internet helps citizens prepare for and respond to heat events		-2.5		3.30
29. Access to television helps citizens prepare for and respond to heat events	-1.67	1.6	2.29	2.55
		-0.75		1.91
30. Access to mobile phone helps citizens prepare for and respond to heat events	0.56	-1.2	2.88	2.19
		-0.75		3.40
31. Access to good quality water is important in maintaining health during heat	-1.44	-1.2	1.59	2.05
		-1.75		0.96
32. Communities where people have good relations are less vulnerable to heat	0.67	-1.4	2.50	2.41
		-0.25		2.63
33. Communities with patrol teams can counter heat risks from living alone	1.56	1.8	1.33	1.30
		1.25		1.5
34. People living in communities where there is more participation in decision-making may be better connected and so at less risk from heat	0.44	1.2	3.24	3.56
		-0.5		3
35. People who are homeless are at greater risk	-1.56	1.4	1.74	2.07
		-1.75		1.5
36. Good access to transportation can help people to avoid or reduce heat risk	-0.56	-0	2.19	2.24
		-1.25		2.22

Table 4: individual Q-Sort rankings for disaster management professionals

Statement	Average Rank	Standard Deviation
1. Good access to medical facilities (hospitals, clinics) mitigates against heat risk	0.5	1.29
2. People working in construction are vulnerable to heat	1.75	1.5
3. People working in farming and fisheries are vulnerable to heat	2	1.73
4. People working outdoors or in manual work are at particular risk	2.75	1.26
5. Elderly people (over 65 years) are at great heat risk	1.5	1
6. Elderly people (over 75 years) are at great heat risk	2.75	1.89
7. Young people (under 15 years old) are vulnerable to heat	-0.75	0.5
8. Very young people (under 5 years old) are vulnerable to heat	1.75	2.87
9. People who have pre-existing chronic health conditions are at greater risk in heat	0.75	2.22
10. People with pre-existing circulatory diseases are at greater risk in heat	0.75	1.71
11. People with pre-existing respiratory diseases are at greater risk in heat	1	1.83
12. People with mental health problems may be subject to additional stress during extreme heat	-1	2.16
13. People with an unhealthy lifestyle (e.g. tobacco smoking, alcohol drinking) are more vulnerable to heat	0	1.41
14. Gender affects vulnerability to heat	-3.5	1.73
15. People who belong to an indigenous group are more vulnerable to heat	-3	2.45
16. People with higher incomes are less vulnerable to heat	0	2.94
17. People living in low-income neighbourhoods are more vulnerable to heat	0.25	2.63
18. Having access to an air conditioner reduces heat vulnerability	4.25	1.5
19. People living in neighbourhoods with greater inequality are more vulnerable to heat	-0.5	1.29

20. Living with family reduces risk in heat	-0.5	1.29
21. Having family nearby reduces risk in heat	-1.75	1.26
22. People who are living alone have greater heat risk	-1.25	0.96
23. Elderly people living alone are at risk in heat	1	1.83
24. People with higher levels of education (university and above) are less vulnerable to heat	-1.75	1.89
25. It is important to understand the local language, otherwise you may not know about heat risk	-0.5	1.73
26. Migrants from overseas who have newly arrived may be at additional risk compared to the cnative population (e.g. language and cultural differences, climate adaptability)	-0.5	2.65
27. Migrants from elsewhere in Taiwan who have newly arrived may be at additional risk compared to the city's native population (e.g. lack of familiarity with community)	-0.5	2.65
28. Access to internet helps citizens prepare for and respond to heat events	-0.5	2.65
29. Access to television helps citizens prepare for and respond to heat events	0.25	1.71
30. Access to mobile phone helps citizens prepare for and respond to heat events	-1	2.94
31. Access to good quality water is important in maintaining health during heat	0.25	1.5
32. Communities where people have good relations are less vulnerable to heat	-0.5	3.32
33. Communities with patrol teams can counter heat risks from living alone	-2.5	1.29
34. People living in communities where there is more participation in decision-making may be better connected and so at less risk from heat	1	3.16
35. People who are homeless are at greater risk	-1.25	1.5
36. Good access to transportation can help people to avoid or reduce heat risk	-0.5	2.38
